# Special Issue: Fungal Cell Wall

**DOI:** 10.3390/jof4030091

**Published:** 2018-08-04

**Authors:** Anne Beauvais, Jean-Paul Latgé

**Affiliations:** Unité des Aspergillus, Institut Pasteur, 75015 Paris, France; jplatge@pasteur.fr

## 1. The Cell Wall of the Ascomycetous Moulds and Yeasts

**Figure 1:**
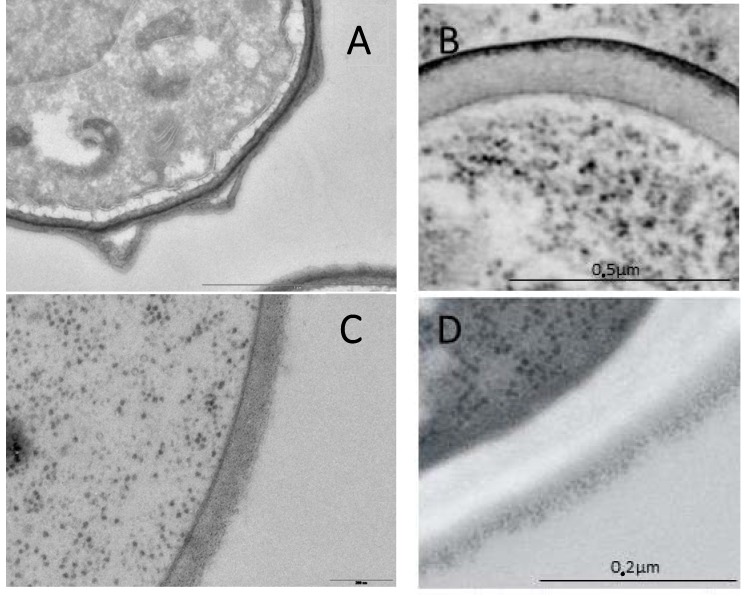
Cell wall from *Ascomycetes*. (**A**) *A. fumigatus* asexual spores; (**B**) *S. cerevisiae* ascopore; (**C**) *A. fumigatus* mycelium; (**D**) *S. cerevisiae* yeast.

The biological cycle of ascomycetous species is characterized by two types of propagules. Spores are the survival morphotypes. Asexual conidia and sexual ascospores allow the fungi to persist under aggressive environments. They are also responsible for the propagation of the species in new territories. The second morphotype is the vegetative form of the fungus, the mycelium, or the yeast which allows the fungus to grow in a nutrient-rich environment where a spore or yeast landed. A characteristic of all the fungal cells is to be surrounded by a cell wall. If the major structural cell wall polysaccharides (β-1,3 glucan and chitin) remain the same on both morphotypes in almost all the *Ascomycete* species, major differences between the two morphotypes can be also observed at the cell wall, mainly related to their different roles in the biological cycle (Figure 1). 

Spores are characterized by a protective layer on the surface of the cell wall. This layer is the first one to come in contact with the environment and protects the spores against a myriad of environmental aggressions such as UV, temperature, high salt, low or high pH, high or low osmotic pressure, and antifungal molecules from the host cells for pathogenic fungi. This layer is also a barrier whose permeability changes overtime. Impermeable to influx of ions, water, and most antifungal chemicals in a resting stage, spores become permeable after germination consecutively to the disruption of this protective layer. During vegetative growth, the cell wall is then reorganized and exposes either fibrillar or amorphous polysaccharides on its surface. Some of the fibrillar cell wall polysaccharides are common to all species—β-1,3 glucan, and chitin—and others—β-1,3-1,4- or β-1,6-glucans, galactomannan, α-1,3 glucan, and mannoproteins—are species-specifics [1]. In addition, a few polysaccharides such as the galactosaminogalactan, mannans and β-1,3 glucans are also specifically secreted and play an essential role during biofilm formation [2,3,4,5,6]. One major finding in recent years is the fact that the cell wall is not a frozen structure but is continuously remodeled when the environment changes [7]. Moreover, even though this has been poorly investigated, the composition of the cell wall changes with the ageing of the cells: at the emergence of the conidial germ tubes or yeast budding or during branching of hyphae, structural alkali-insoluble polysaccharides are degraded, and a new set of these polysaccharides are synthesized and remodeled to maintain a plastic cell wall at the apex. In subapical regions, the same polysaccharides are elongated and branched together to form a network of resistant, inter-connected fibrillous polysaccharides embedded in an amorphous cement of alkali-soluble polysaccharides [8].

This special issue describes the importance and role of the cell wall along the fungal life, from the spore to the vegetative yeast or mycelium, including the synthesis, inhibition and role in fungal infection for pathogenic fungi. This issue is especially focused on three models: the yeasts *Saccharomyces cerevisiae* and *Candida albicans* and the filamentous fungus *Aspergillus fumigatus*, which have accumulated the best cell wall knowledge over the years.

## 2. The Outer Layer of the Cell Wall Is a Protective Barrier

### 2.1. The Ascospores

In *S. cerevisiae*, the outer ascospores cell wall is constituted of chitosan, dityrosine, and an newly identified molecule called “chi” [9]. The chemical structure and the synthesis of chi are still unknown. Chitosan is synthesized through the action of chitin deacetylases (Cda) on the chitin produced by the chitin synthase Chs3. The dityrosine layer is synthesized in the cytoplasm by the enzymes Dit1 and Dit2, which use tyrosine as a precursor. The dityrosine is then transported by the transporters Dtr1 and Dtr2 to the cell wall, where it is assembled into a large polymer by an unknown mechanism. The impermeability and protection of the ascospores require mainly the chi layer and the chitosan. 

### 2.2. Are the Ascospores of S. cerevisiae a Model for Fungal Ascospores?

Ascospores are also found in moulds and especially in *A. fumigatus*, but the composition of their cell wall has been poorly investigated. We have just identified the presence of a high amount of chitosan in their cell wall (A. Neiman and A. Beauvais, unpublished results, 2016). Does this polysaccharide have a function in dormancy establishment or in resistance to external aggressions? It should be the focus of many studies for the future.

### 2.3. The Conidiospores

In *A. fumigatus*, the conidial cell wall is covered by rodlets, constituted by the class I hydrophobin RodA, and by DHN-melanin. These two layers are responsible for the hydrophobicity of the conidium and its resistance to physical disruption, UV, and desiccation [10]. Other classes of hydrophobins have been identified in *A. fumigatus*, but their role remains unknown since they were not able to assembly into rodlets [10]. Rodlets and melanin play a crucial role in protecting the conidium against host immune cells. The rodlets immunosilence the conidium and prevent the activation/maturation of dendritic cells, macrophages, or neutrophils which do not produce cytokines upon contact with the conidia [11,12]. However, conidia are recognized by host soluble mediators and, through the activation of the major complement component C3, opsonize the conidia, enhancing phagocytosis by macrophages through the binding of the immune-complex to receptors present on the immune cells [13]. Activation of the complement could result from one or more of three different pathways: alternative, classical, and lectin pathways, activated by the binding of distinct pattern-recognition molecules on the conidial surface. In addition to complement components, another group of soluble mediators, SP-A, SP-D and pentraxin-related protein 3 (PTX3), are also able to bind and opsonize conidia, facilitating phagocytosis (Sze17). PTX3 is known to be a major facilitator of aspergillus invasion [14]. Instead of hiding the fungus from the host cells like the rodlet layer, the melanin plays a direct antifungal role by inhibiting conidial killing within the phagosome by blocking phagosome biogenesis, acidification, and NADPH organization [15]. 

Are the conidia of *A. fumigatus* a model of asexual spores? The rodlet layer has been studied previously in the conidia of other moulds such as *Neurospora*, *Penicillium*, and *Cladosporium* [11,16]. Rodlet organization and immune inertia has been also reported for other species like for *A. fumigatus* rodlets. However, no further studies were undertaken on the conidia of these different species.

### 2.4. The Germinated Conidia

The germination of the conidia has been mainly studied in *Aspergillus* species (*A. fumigatus* and *A. nidulans*). As soon as the conidia of *A. fumigatus* are in presence of nutrients and favorable conditions, the protective external layers are disrupted, exposing the inner polysaccharides α-1,3 glucan, galactomannan, and GAG neosynthesized during early stages of germination. These surface components have a protective role for pathogenic species because they hide polysaccharides which are more immunostimulatory, such as β-1,3 glucan and chitin recognized by dectin-1 and IgG, respectively. However, they can also have an essential immune role which facilitates fungal invasion [17,18].

#### 2.4.1. The α-1,3 Glucan

Aggregation of swollen *A. fumigatus* conidia and hyphae during germination and biofilm formation results from interactions of α-1,3 glucan chains between themselves. The repeating unit of α-1,3 glucan is composed of two linear α-1,3 glucans linked by an α-1,4 oligosaccharide. Even though this polysaccharide is essential not only for cellular aggregation but also for growth, cell wall resistance and virulence by hiding β-1,3 glucan exposition [19,20,21], its biosynthesis is not fully understood. To date, it is only agreed that the biosynthesis of the α-1,3 glucan chains results from the activity of α-1,3 glucan synthases (Ags), whose number depends on the species [20]. However, in all the species producing this polysaccharide, α-1,3 glucan is synthesized mostly by one major Ags. The synthesis of the α-1,4 glucan is also controversial. Resulting from the activity of Ags1 in *S. pombe* [21], the α-1,4 glucan synthesis involves amylases coded by *AMY* genes localized in the same cluster as *AGS* in *A. orizae* (*AMYG*-*AMYB*-*AGSB*), *A. niger* (*AMYE*-*ATGA*-*AGSE*), and *A. fumigatus* (*AMY1*-*AGS1*) [19]. The substrate for α-1,3 glucan synthesis is still unknown, but it is assumed that an α1,4 glucan oligosaccharide, first synthesized, could serve as a primer for α-1,3 glucan chain initiation. 

#### 2.4.2. The GAG

The GAG is a heteropolysaccharide composed of galactopyranose, N-acetylgalactosamine, and galactosamine residues. GAG is synthesized as soon as the conidia start germinating [22]. Its synthesis is under the control of a gene cluster of five co-regulated genes *GTB3*, *AGD3*, *EGA3*, *SPH3*, and *UGE3* [23]. GAG has strong, multifunctional adhesive properties [24] and is a virulence factor, inducing neutrophil apoptosis, resistance to neutrophil extracellular traps, and an immunomodulatory function by inducing IL-1Ra [17,18,25].

#### 2.4.3. The Mannans

In yeast, mannan interactions are involved in the formation of biofilms. Families of mannan-bearing adhesins are known, such as FLO in *S. cerevisiae*, EPA in *C. glabrata*, and ALS in *C. albicans* [5,26,27]. The long mannan chains are synthesized in yeasts by the α mannosyltransferase complex I and II, localized in the endoplasmic reticulum and Golgi vesicles. In *A. fumigatus*, the synthesis of the mannan moiety of *A. fumigatus* galactomannan follows another pathway that is still unknown [28]. The deletion of the 11 genes which are putative orthologs of the yeast mannosyltransferases did not lead to a reduction of the mannan content of the cell wall of the mycelium of *A. fumigatus*. In contrast, the mannan content of the conidial cell wall was reduced, and this reduction was associated with a partial disorganization of the cell wall leading to defects in conidial survival both in vitro and in vivo.

## 3. Biosynthesis of the Cell Wall Fibrillar Core

Synthesis of the β-1,3 glucan and chitin, which are the most important polysaccharides of the mature cell wall because they represent the skeleton of the cell wall, is obviously initiated at the plasma membrane. Synthases at the plasma membrane responsible for the synthesis of linear polysaccharides have been well identified [1]. The biosynthetic enzymes β-1,3 glucan, and chitin synthases are transported to the plasma membrane by the cytoskeleton and are part of vesicles forming the spizenkorper [29]. A turn-over has been observed, with the enzymes going back into vesicles to be degraded. 

The remodeling enzymes which are essential in the construction of the 3D scaffold characteristic of the cell wall are also associated to the cell wall membrane. How all these membrane proteins are associated and interfering have not been fully elucidated. It was recently found that glucanases, chitinases, branching and elongating enzymes are present in membrane compartments of the H+ ATPase Pma1 (MCP) which are active dynamic domains [30]. Many of these cell wall synthases and remodeling or degrading enzymes are attached to the membranes via a glycosylphosphatidylinositol (GPI). The localization of the MCP is transient and under the control of another type of membrane compartment of the arginine permease Can1 (MCC), associated to cytoplasmic proteins that form the eisosomes complex. The MCC/eisosomes are immobile and stable patches in the plasma membrane [30]. The GPI lipidic anchor of the remodeling enzymes allows them to face the outer layer of the membrane. The GPI biosynthesis pathway occurs in the endoplasmic reticulum where the anchor is transferred to the protein. This pathway is essential in all eukaryotes including fungi [31]. In *A. fumigatus*, the GPI-proteins have at least four main roles in cell wall biosynthesis: (i) Elongating of β-1,3 glucan with GEL/GAS/PHR enzymes, which belong to the GH72 family. In this family, some GPI-enzymes possess the carbohydrate binding module of family 43 (CBM43, GH72^+^ enzymes) essential for their function in the essential genes; (ii) Branching of β-1,3 glucans together through β1,6 linkages with BGL/BGT family which belong to the GH17 family. Cooperation for branching between GH17 and GH72^+^ enzymes has also been demonstrated; (iii) β-1,3 glucan-chitin reticulation with the CHR family; and (iv) β-1,3 glucan-galactomannan cross-linking with the DFG family. Given the impact of these families on the cell wall and the importance of the cell wall structure in mediating host-pathogen interactions, these families are essential for virulence and in some instances even for fungus viability. The latter is observed for Gel4 in *A. fumigatus* [32], Gas1 and Gas2 [33], and Phr1–Phr2 in *C. albicans* [34].

Other cell wall remodeling enzymes are not GPI-proteins. However, some of them belong to the CAZYME families mentioned above such as the glycosyltransferase Bgt1/Bgl1, Bgt2/Bgl3, or the putative β-1,3 glucanases Scw4 and Scw11, all from family GH17 [35]. Other β-1,3 glucanases belonging to families GH16 and GH81 are involved in *A. fumigatus* conidial chain separation [36]. 

Cell wall biosynthesis appears then as a dynamic, essential, and timely process that is correlated with growth. It has been accepted that the construction of the cell wall results from an equilibrium between synthesis and hydrolysis [37]. The story is far from being finished.

## 4. Adaptation of the Cell Wall to Environmental Conditions and Cell Wall Drugs

The cell wall is the first fungal organelle interacting with the environment. It responds to every stress signals. The cell wall integrity signaling (CWI), the high-osmolarity glycerol (HOG), and calcineurin pathways provide maintenance of cellular integrity and fungal survival. 

The response to stress involves a sensing apparatus, localized at the plasma membrane that activates signaling pathways. According to the type of stressor, the sensor will be different and consequently the signaling pathway also. Hk1, the sensor of the β-1,3 glucanase zymolyase, activates the HOG signaling pathway while Wsc1, the echinocandin sensor, and Mid1, the congo red sensor, activate the CWI pathway [34,38]. However, these two pathways have in common the activation of Pkc1-Mkc1 kinase pathway which finally activates transcription factors. Rml1 is the transcription factor responsible for the expression of the majority of the genes induced under cell wall stress [38]. The calcineurin pathway is activated following a modification of the intracellular Ca^2+^ concentration due to stretching or warping of the plasma membrane associated to cell wall stress [34]. Ca^2+^ is a crucial second messenger in eukaryote cells. The calcineurin pathway activates Crz1 transcription factor, inducing cell wall reorganization. In addition to cell wall remodelling, the signaling pathways also induce the regulation of expression and production of specific clusters leading to the production of secondary metabolites such as mycotoxins or the pigment melanin [39], which are used by *A. fumigatus* to resist to different biotic stresses due to bacteria or phagocytes. 

The general effect of the activation of the CWI, HOG, or calcineurin pathways is the increase of chitin level in the cell wall. This effect has been extensively studied in response to echinocandin exposure. Echinocandin inhibits β-1,3 glucan synthase (GS) activity by binding on localized plasma membrane outer domains of the enzyme—the hot spots [40,41]. Following incubation of the fungus with the drug, altered GS is internalized and degraded intracellularly, whereas chitin synthase is upregulated, following the activation of the CWI pathways. However, another fungal response has been described in *C. albicans* and *A. fumigatus* after exposition to high doses of caspofungin and other echinocandins—paradoxical growth [42]. During paradoxical growth, the fungus became resistant to echinocandins. This phenomenon is correlated with the increase of chitin level and the reconstitution of the GS activity. In *A. fumigatus*, the chitin level of paradoxically hyphae returns to normal, and the newly synthesized GS is protected against echinocandin by an unknown mechanism.

## 5. Conclusions

Cell wall structural cores are identical or very similar in the different morphotypes of ascomycetous yeast and moulds. Accordingly, it could be expected that the molecular mechanisms regulating the synthesis of the essential polysaccharides would be the same. However, they vary between species. For example, in the budding yeast, two GPI biosynthetic pathways have been proposed: a main sequential pathway is via the enzymes Per1, Cwh43, and Gup1 and an alternative minor pathway in which Cwh43 can use different substrates [31,43]. In *A. fumigatus*, the alternative pathway is absent [31]. Variations are even more important for the non-structural polysaccharides or the remodeling of the 3D cell wall. For example, the biosynthesis of mannans is very different in yeasts and moulds in spite of very homologous mannosyltransferases with similar in vitro activity [28]. Similarly, another example (not discussed here) is the chitin synthases of yeasts and moulds [44,45]. In silico similarities does not indeed mean always identical function in different species, and it is clear that orthologous genes code for proteins that have very different functions in yeasts and filamentous fungi. It also indicates that the important function of cell wall enzymes cannot be separated to the overall cell wall organization. 

Cell wall structure has been discussed for the last 50 years. Yet, so little is known despite the fact that it plays a key role in fungal growth and survival. We hope that this editorial and specific issue on the cell wall will stimulate new career interests, even though the new volunteers will have to deeply enter the difficult fields of carbohydrate chemistry. Study on the biosynthesis of the fungal cell exoskeleton will also be of the greatest value to understand key biological issues such as polarity establishment. Moreover, on the applied side, polysaccharide components of the cell wall are unique to fungi, and, consequently, putative inhibitors of the biosynthetic pathways responsible for cell wall construction such as the echinocandins are therefore unlikely to have secondary toxic effects. New drugs are always required due to the quick emergence of resistance when a new pesticide is launched. Identifying such antifungal molecules will help both health and agriculture since fungi, even though they are not regarded as key pathogens, are one of the most detrimental, and clinical costs can exceed 100,000 Euros per human infection.
